# Optimization strategies for public health education based on ISSA and information system technology

**DOI:** 10.3389/fpubh.2025.1523876

**Published:** 2025-05-08

**Authors:** Zhanyu Ye, Yifei Li, Yan Zhang

**Affiliations:** ^1^School of Population Health & Environmental Sciences, King’s College London, London, United Kingdom; ^2^Department of Industrial Engineering and Operations Research, Columbia University, New York, NY, United States; ^3^Judge Business School, Cambridge University, Cambridge, United Kingdom

**Keywords:** public health education, ISSA, information systems technology, resource optimization, educational effectiveness

## Abstract

**Introduction:**

Against the backdrop of rapid development of information technology, public health education is facing challenges such as uneven resource allocation and lagging content.

**Methods:**

To propose an optimization strategy that can effectively improve the level of public health education, this study improves the sparrow search algorithm by introducing the theory of best point sets to optimize the allocation of public health education resources. Combined with information system technology, a public health education information platform is proposed to optimize public health education.

**Results:**

The experiment findings denoted that the improved sparrow search algorithm had a significantly better average fitness value than other compared algorithms after 500 iterations, with an accuracy of 92.4% and an area under the PR curve of 0.84. In practical application, the optimization model for public health education resources increased the balance of resource allocation to 0.89, improved educational effectiveness by 25.5%, and increased user satisfaction by 31.4%. At the same time, the constructed public health education information platform showed excellent performance in terms of CPU usage and time consumption, significantly improving education coverage and content update frequency.

**Discussion:**

The above findings indicate that the optimization strategy raised in the study provides scientific basis and practical guidance for the optimization of public health education, which helps to raise the effectiveness and quality of public health education.

## 1 Introduction

In today’s rapidly developing information technology, public health education, as an important link in ensuring public health and preventing disease transmission, is particularly important for optimization and improvement (1). However, current public health education is facing many challenges, such as uneven distribution of educational resources, lagging educational content, and a single educational model (2). These issues not only affect the effectiveness of public health education, but also constrain its role in responding to sudden public health emergencies (3). Recently, with the rapid growth of intelligent optimization algorithms and information system technology, new ideas and methods have been provided for the optimization of public health education (4). Among them, the Improved Sparrow Search Algorithm (ISSA), as an emerging swarm intelligence optimization algorithm, has demonstrated significant merits in multiple fields due to its fast convergence speed and strong global search ability. For example, Israil et al. designed a traffic prediction framework based on an improved ISSA algorithm to address network congestion and latency issues in medical system data transmission, in order to improve network response efficiency. Its multi-step prediction is superior to traditional methods (5). In the field of disease detection, Wazery et al. utilized an improved ISAM algorithm based on ISSA reverse learning strategy to collect and process large amounts of medical data more quickly, and combined it with K-nearest neighbor classifier to propose a new classification model. Compared with other models, its classification accuracy has been significantly raised (6). The Yu team proposed a brain disease classification method based on ISSA to raise the accuracy of diagnosing brain diseases using image features, and introduced three improvement mechanisms to enhance accuracy. The outcomes denoted that the classification accuracy of the ISSA-KNN model exceeded 85%, which was superior to other algorithms and classifiers (7).

Information system technology provides more accurate and efficient support for public health education through data collection, analysis, processing, and other means (8). For example, Matloob et al. designed an intelligent fraud detection model based on sequence mining to address the surge in fraudulent medical transactions. After verification with real data, the model was highly applicable in complex doctor-patient relationships, and experimental results showed that it could detect and filter a higher number of fraud cases than other models (9). Gong and his team proposed a private cloud-based information system resource management method to address the significant increase in computing resource load of medical data. By utilizing intelligent control theory to dynamically adjust resource allocation, the method effectively reduced IT costs by over 23% in experimental simulations (10). Mudawi et al. proposed an intelligent ICU patient monitoring system to optimize the monitoring process of real-time detection of patients’ physical signs and timely feedback of abnormal data. The system can monitor patients’ blood pressure, heart rate, blood oxygen, etc. in real time, improving the efficiency of ICU patient management by more than 15% and reducing the workload of doctors (11). Although the application of ISSA and information system technology in public health education has made some progress, there are still some shortcomings in current research. For example, the specific application of ISSA in the field of public health education is not yet sufficient. How to combine it with the actual needs of public health education, optimize the allocation of educational resources, and accurately push educational content is still an urgent problem to be solved. Meanwhile, the application of information system technology in public health education also faces challenges such as uneven data quality and difficulty in information sharing (12).

Based on the above research background and current situation, the research aims to explore optimization strategies for public health education based on ISSA and information system technology. Introducing the ISSA can optimize the allocation of public health education resources and accurately deliver educational content, thereby improving education efficiency and quality. Meanwhile, utilizing information system technology, a public health education information platform is constructed to collect, analyze, process, and share educational data, providing data support for the optimization of public health education. The innovative point of the research is to combine the theory of best advantage set and ISSA to optimize resource allocation, which provides a new framework; By introducing the optimal set theory, the efficiency of the optimization algorithm in resource search process is improved, and the local optimal trap that may exist in the existing algorithm in the complex allocation problem is overcome, so as to achieve higher global search capability. Construct an integrated information system technology platform to support the collection, analysis, processing and sharing of public health education resources. The platform can accurately push educational content and feedback users’ needs in real time to further improve the utilization efficiency of resources and the pertinency of education.

## 2 Methods and materials

### 2.1 An ISSA introducing the theory of optimal point sets

**Figure 1:**
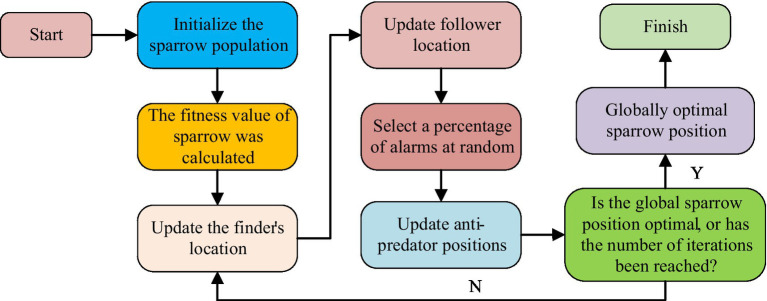
Workflow of SSA.

Since the powerful global search capability and fast convergence characteristics of SSA algorithm, these features have significant advantages in solving problems such as uneven allocation of public health education resources and lagging educational content (13–15). Therefore, this study introduces the SSA in the optimization of public health education, hoping to utilize its advantages to raise the development of the field of public health education. The SSA is a swarm intelligence optimization algorithm that simulates the foraging behavior of sparrows. Its core idea is to achieve global search of the problem space by simulating the cooperative and competitive behavior of sparrows in searching for food. The workflow of this algorithm is shown in Figure 1 (16–18).

In Figure 1, during the initialization phase of the SSA, the sparrow population is randomly distributed in the search space, with each sparrow representing a potential solution. The initialization position formula of sparrow population is denoted in Equation 1 (19).

(1) $$X_{i}^{d}=\mathrm{rand}\left(\right)\times \left(ub-\mathrm{lb}\right)+\mathrm{lb}$$

In Equation 1, $X_{i}^{d}$ denotes the position of the i th sparrow in the d th dimension, $\mathrm{rand}\left(\right)$ denotes the random number within the interval $\left[0,1\right]$ , ub , and lb mean the upper and lower boundaries of the position, respectively. Next, the algorithm enters the iterative process and evaluates the quality of the solution corresponding to its position by calculating the fitness value of each sparrow. The fitness calculation expression is shown in Equation 2.

(2) $$f\left(X_{i}\right)=\mathrm{ObjectiveFunction}\left(X_{i}\right)$$

In Equation 2, $f\left(X_{i}\right)$ denotes the fitness value of the i th sparrow, and $f\left(X_{i}\right)$ represents the objective function. According to their fitness values, sparrows are classified into three categories: discoverers, followers, and scouts, and their positions are updated accordingly. The discoverer is responsible for searching and sharing food information within a local area, and their location update expression is denoted in Equation 3.

(3) $$X_{i}^{d,\mathrm{new}}=X_{i}^{d}+\Delta X$$

In Equation 3, ΔX represents the amount of position update. In addition, the follower adjusts their position based on the discoverer’s information, while the scout searches for new food sources globally. The scout’s position update expression is denoted in Equation 4.

(4) $$X_{i}^{d,\mathrm{new}}=X_{i}^{d}+\mathrm{rand}\left(\right)\times \left(X_{\mathrm{best}}^{d}-X_{i}^{d}\right)$$

**Figure 2:**
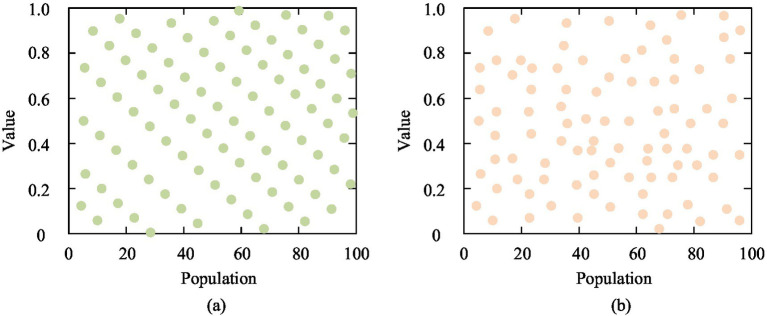
Comparison between random initialization and optimal point set initialization.

In Equation 4, $X_{\mathrm{best}}^{d}$ represents the position of the current optimal solution in the d th dimension. However, the original SSA may have limited optimization performance when dealing with complex optimization problems due to getting stuck in local optima (20, 21). To overcome this limitation, the study introduces the theory of optimal point sets based on the SSA, forming the ISSA, to achieve more accurate and efficient resource allocation and content push in public health education optimization. Among them, the optimal point set theory is a mathematical method used to optimize the search process, which improves search efficiency by identifying and prioritizing areas in the search space that are more likely to produce high-quality solutions (22, 23). Compared with the random initialization method, the optimal point set initialization method is more targeted, and the search comparison between the two methods is shown in Figure 2 (24).

In Figure 2, random methods usually distribute search points uniformly without considering the possibility of producing high-quality solutions at each point. The optimal point set method predicts and selects the regions that are more likely to find the global optimal solution by analyzing the characteristics of the problem and historical search data. The optimal point set method aims to optimize a given objective function by selecting or updating a set of points, where the expression for initializing the optimal point set is shown in Equation 5.

(5) $$X_{i}^{0}=\mathrm{BestPo}int\mathrm{Set}\left(f,N\right)$$

Equation 5 represents selecting an initial set of optimal points $X_{i}^{0}$ through the function BestPointSet at the beginning of the iteration process. This function selects the optimal point set based on the objective function f and the number of initial points N . Afterwards, it will iterate to update the optimal point set, and the specific formula for updating the best point set is shown in Equation 6.

(6) $$X_{i}^{t+1}=X_{i}^{t}+\alpha \cdot \left(\mathrm{BestSe}t^{t}-X_{i}^{t}\right)$$

**Figure 3:**
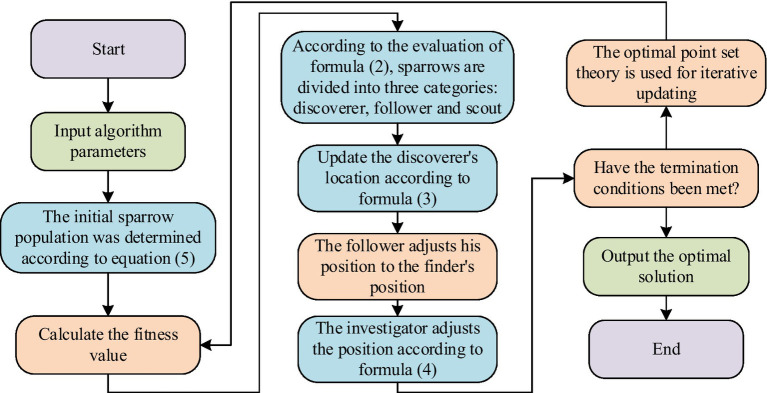
Specific flow of ISSA.

In Equation 6, t represents the number of iterations, $X_{i}^{t+1}$ and $X_{i}^{t}$ represent the best point set for the next iteration and the current best point set. α is the learning rate parameter, which controls the step size towards the current optimal point set $\mathrm{BestSe}t^{t}$ . The specific process of ISSA obtained by combining the theory of optimal point set with SSA is shown in Figure 3.

From Figure 3, firstly, ISSA performs the initialization stage, using the optimal point set theory to select the initial sparrow population position, rather than the traditional random distribution. Using the function selection method in Equation 5, based on the objective function and the number of initial points, a more promising initial set of optimal points is selected as the starting position for the sparrow population. Next is entering the iterative process. The fitness value of each sparrow is calculated and the quality of the solution corresponding to its position is evaluated based on Equation 2. According to fitness values, sparrows are divided into three categories: discoverers, followers, and scouts. The discoverer updates the location based on Equation 3, searches and shares food information within the local area. Followers adjust their position based on the discoverer’s information, while scouts search for new food sources globally according to Equation 4 to escape local optima. In the iterative process, the optimization mechanism of the best point set theory is introduced, and the best point set is iteratively updated through Equation 6. By continuously optimizing the optimal point set, the sparrow population is guided to move towards areas that are more likely to produce high-quality solutions, improving search efficiency. After reaching the preset number of iterations or satisfying convergence conditions, the algorithm outputs the optimal solution, which is the best plan for allocating public health education resources and content push.

### 2.2 Optimization model of public health education resources based on ISSA

**Figure 4:**
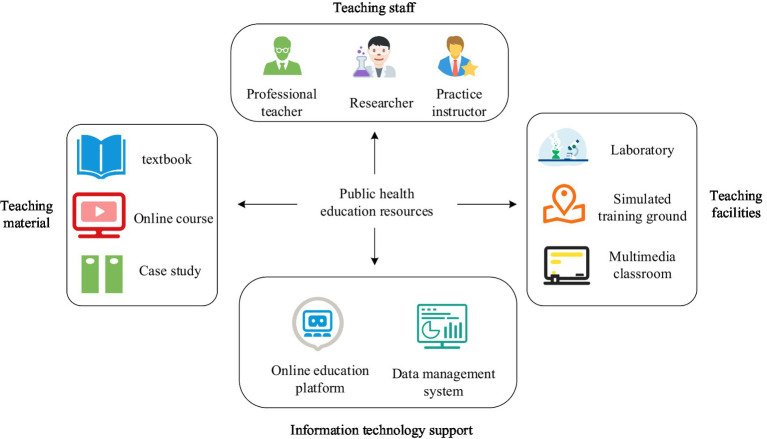
Main classification of public health education resources.

The optimization of public health education mainly relies on the optimization of educational resources, which support the improvement of education quality and efficiency, thereby enhancing the capacity of public health services. The two are closely connected and jointly promote the development of public health (25, 26). However, there is currently a challenge of uneven distribution of public health education resources, and innovative optimization methods are particularly important. The ISSA proposed in the study provides new ideas for optimizing the allocation of public health education resources by simulating the collaboration and competition mechanisms of sparrow populations during foraging. Among them, public health education resources are an important support for the public health education system, covering a wide range of content, and their main classification framework is shown in Figure 4 (27, 28).

In Figure 4, public health education resources mainly include but are not limited to teaching materials (such as textbooks, online courses, case studies, etc.), teaching staff (professional teachers, researchers, and practical guides), teaching facilities (laboratories, simulation training venues, etc.), and information technology support (online education platforms, data management systems). The rational allocation and efficient utilization of these resources are directly related to the quality and efficiency of public health talent cultivation, which in turn affects the overall effectiveness of public health services. Combining these different resource types into a single parameter is not appropriate because the characteristics and usage of each resource are significantly different. Certain resources, such as online teaching materials and information technology support, can be shared and accessed remotely through the network to ensure flexibility and availability of resources; Other resources, such as laboratories and field training facilities, can only be used offline in specific locations, limiting their shared reach and efficiency. Therefore, for the optimization of public health education, it is necessary to formulate independent optimization strategies for various resources to ensure reasonable and efficient allocation of various resources and maximize the educational effect. In the optimization model, considering the characteristics of different resources can more accurately reflect the actual needs, thus improving the overall effectiveness and quality of public health education. Let R represent the total resources, Teaching materials, teaching staff, teaching facilities and information technology are represented by $R_{1}$ , $R_{2}$ , $R_{3}$ , and $R_{4}$ respectively. $A_{a}$ represents the a th region, institution, or project, $x_{a}$ represent the proportion of resources allocated to $A_{a}$ , and the goal is to find a set of $x_{a}\left(a=1,2,\cdots n\right)$ that maximizes a certain objective function $f\left(x_{1},x_{2},\cdots x_{n}\right)$ . This objective function can represent educational outcomes, public health service capabilities, etc., with the specific expression shown in Equation 7.

(7) $$\left\{ \begin{array}{l} \max f\left(x_{1},x_{2},\cdots ,x_{n}\right)=\sum_{a=1}^{n}x_{a}\cdot E_{a}\\ s.t.\sum_{a=1}^{n}x_{a}=1,x_{a}\geq 0,\sum_{a=1}^{n}x_{a}^{2}\leq \delta^{2} \end{array}\right.$$

In Equation 7, $E_{a}$ represents the efficiency of the a th region, institution, or project, and $\delta^{2}$ is the upper limit of the variance of the allocation strategy to ensure the stability of resource allocation. Variance is an index that describes the degree of dispersion of a set of data distributions. A small variance means that the data distribution is concentrated and the distribution results are more uniform. By setting the upper limit of variance, the extreme allocation of resources between different targets can be effectively limited, so as to avoid the excessive concentration of resources in some regions and the possible shortage of resources in others due to the uneven distribution of resources. This restriction helps to form a relatively balanced resource allocation pattern and enhance the accessibility and equity of education services. The stability of resource allocation is also reflected in the promotion of resource use efficiency. The effective use of resources often depends on the synergy between various resources. If the distribution of resources fluctuates too much, it may lead to the reduction of the efficiency of use, and then affect the effect of education. For example, when teaching staff is too concentrated in a certain region and teaching facilities are relatively scarce, although the teaching quality in the region may be improved, the overall effect of public health education may not be improved, but may affect the level of education services in other regions. Faced with the challenges of limited and uneven distribution of public health education resources, innovative optimization methods are particularly important. Under the ISSA framework proposed in the study, each sparrow is considered as a potential resource allocation scheme, with its position $X_{j}=\left(x_{j1},x_{j2},\cdots ,x_{jn}\right)$ representing the allocation ratio of resources in different regions, institutions, or projects, where j represents the sparrow’s index. The algorithm iteratively updates the position of sparrows, continuously adjusting the resource allocation strategy to approach the optimal solution. Specifically, the ISSA incorporates the theory of optimal point sets, which calculates the fitness value of each sparrow position and preserves the sparrow positions with higher fitness, guiding the search process towards the global optimum direction. Assuming P represents the best point set and $P_{\mathrm{best}}$ represents the current best position, the updated formula is shown in Equation 8.

(8) $$X_{j}^{t+1}=X_{j}^{t}+\alpha \cdot \left(P_{\mathrm{best}}-X_{i}^{t}\right)+\beta \cdot \left(\mathrm{rand}\left(\right)-0.5\right)$$

**Figure 5:**
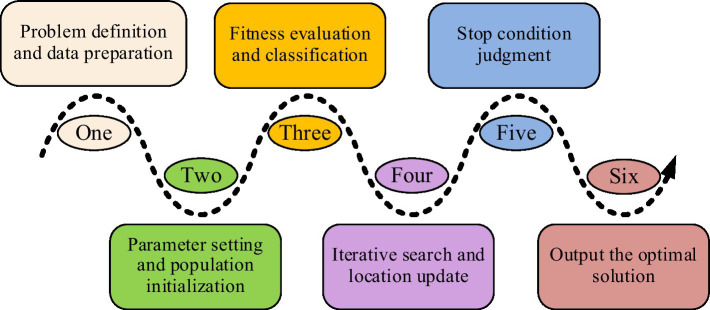
Operation flow of public health education resource optimization model.

In Equation 8, α and β are control parameters. Through a series of iterations and optimizations, the ISSA can efficiently explore the optimal solution for resource allocation, providing scientific basis for the rational allocation of public health education resources. Therefore, the study proposes a public health education resource optimization model based on the ISSA, hoping to better optimize public health education through this model. The operational process of the public health education resource optimization model proposed in the study is shown in Figure 5.

In Figure 5, the proposed public health education resource optimization model mainly includes six steps. Step one is to clarify the optimization objectives, collect and organize relevant data on public health education resources. Step two is to set the parameters of the ISSA, such as population size, iteration times, learning rate, etc., and initialize the sparrow population position using the optimal point set theory. Step three is to calculate the fitness value of each sparrow and classify them into three categories: discoverer, follower, and scout based on their fitness value. When calculating the fitness value of each sparrow, a specific objective function is needed to evaluate the effectiveness of the resource allocation plan. Let $f\left(X_{j}\right)$ denote the fitness value of the j th sparrow (i.e., the j th resource allocation plan), which can be a quantitative indicator of educational effectiveness, public health service capacity, etc. The objective function can be indicated as Equation 9.

(9) $$f\left(X_{j}\right)=\overset{n}{\underset{a=1}{\sum }}\omega_{a}\cdot g_{a}\left(xj_{a}\right)$$

In Equation 9, $\omega_{a}$ is the weight of the a th region, institution, or project, and $g_{a}\left(xj_{a}\right)$ is a function of the resource ratio $xj_{a}$ , representing the educational effectiveness or public health service capacity of the a th region, institution, or project under this allocation ratio. The next step is to conduct iterative search, where discoverers, followers, and scouts update their positions based on their respective search strategies, that is, adjust the resource allocation plan. For discoverers, they usually update based on the current best location $P_{\mathrm{best}}$ and their own location to explore new resource allocation schemes. The updated formula can be further refined into Equation 10.

(10) $$X_{j}^{t+1}=X_{j}^{t}+\alpha \cdot \left(P_{\mathrm{best}}-X_{i}^{t}\right)+\beta \cdot \left(\mathrm{rand}\left(\right)-0.5\right)\cdot \left(UB-LB\right)$$

In Equation 10, UB and LB mean the upper and lower bounds of the resource allocation ratio, respectively. Equation 10 ensures that the updated position remains within the effective resource allocation range. The fifth step is to determine whether the stopping conditions are met, such as reaching the max amount of iterations or convergence of fitness values. The final step is to output the optimal sparrow position, which is the best allocation plan for public health education resources, after the iteration is completed.

### 2.3 Construction of an education information platform integrating information system technology and ISSA resource optimization model

**Figure 6:**
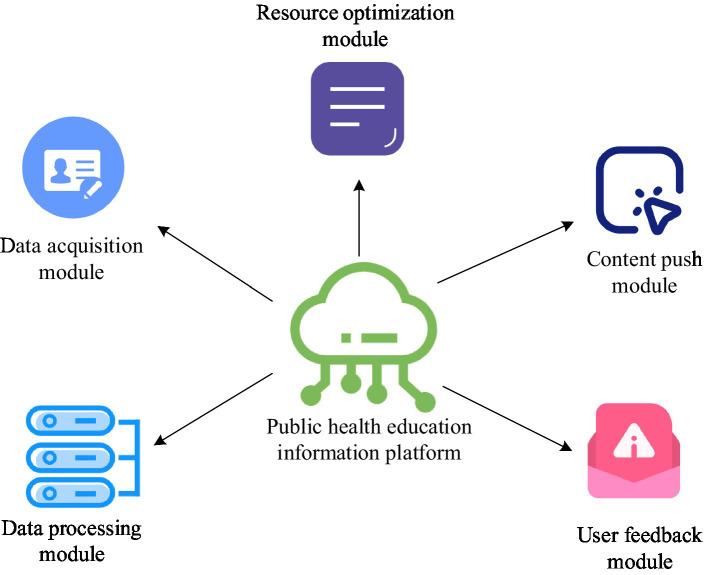
Architecture diagram of education information platform integrating information system technology and ISSA resource optimization model.

Although the ISSA resource optimization model constructed in the study has good optimization effects, there are significant shortcomings in data collection and processing. In public health education, information system technology can achieve the collection, analysis, processing, and sharing of educational data, providing strong support for educational optimization (29, 30). In public health education, information system technology can achieve comprehensive collection and analysis of educational resources, learner information, teaching effectiveness, and other data. Through these data, it can gain a deeper understanding of the current situation and needs of public health education, providing data support for educational optimization (31, 32). To combine the optimization results of ISSA with practical applications, a public health education information platform integrating information system technology and ISSA resource optimization model is developed. The structure of the public health education information platform constructed through research is shown in Figure 6.

In Figure 6, the proposed platform mainly includes data collection module, data processing module, resource optimization module, content push module, and user feedback module. The data collection module is responsible for collecting various educational resources and learning behavior data. In this module, information system technology is used to construct a data collection model, as shown in Equation 11.

(11) $$D_{i}=\sum_{j=1}^{n}S_{j}\cdot T_{k}$$

In Equation 11, $D_{i}$ represents the i th class data, $S_{j}$ represents the data collection source, and $T_{k}$ represents the data collection time. The data processing module cleans, integrates, and transforms data to provide a foundation for subsequent analysis. The resource optimization module utilizes the ISSA to optimize the allocation of educational resources, ensuring that resources can efficiently serve teaching needs. The resource optimization model is shown in Equation 12.

(12) $$\max f\left(x_{1},x_{2},\cdots ,x_{n}\right)\;s.t.\;\begin{array}{ccc} \sum_{i=1}^{n}x_{i}=1 & x_{i}\geq 0 & \forall i \end{array}$$

In Equation 12, $x_{i}$ represents the proportion of resources allocated to the i th region, institution, or project. The content push module accurately pushes educational content based on users’ learning needs and interest preferences, and constructs a content push model as shown in Equation 13 in this module.

(13) $$C_{i}=\sum_{j=1}^{m}U_{j}\times P_{k}$$

In Equation 13, $C_{i}$ represents the i th category of educational content, $U_{j}$ represents the user group, and $P_{k}$ represents the push strategy. This model can accurately push educational content based on users’ needs and interests. The user feedback module is used to collect users’ opinions and suggestions on the platform. In this module, a user satisfaction evaluation model as shown in Equation 14 is adopted to better optimize the platform.

(14) $$S=\sum_{i'=1}^{n}Q_{i'}\times W_{j'}$$

In Equation 14, $Q_{i'}$ represents the quality of the $i^{\prime }$ th service, $W_{j'}$ denotes the weight of user satisfaction, and S represents the user satisfaction score. By constructing a public health education information platform that integrates information system technology and ISSA resource optimization model, it is possible to optimize the allocation of educational resources and accurately push educational content, thereby improving the effectiveness and quality of public health education. At the same time, the platform can also provide users with convenient educational services and learning experiences, promoting the popularization and development of public health education. In summary, the research proposes optimization strategies for public health education based on ISSA and information system technology. Firstly, the ISSA is utilized to optimize the allocation of public health education resources. By collecting and analyzing relevant data on educational resources, a resource optimization model is constructed, and algorithm parameters are set. Then, the iterative search capability of the ISSA is utilized to find the optimal resource allocation plan, ensuring that resources can efficiently serve teaching needs. Secondly, by combining information system technology, it will build a public health education information platform to achieve precise delivery of educational content. Data on learners’ learning behavior and interest preferences are collected through the data collection module, and then the data processing module is used to clean, integrate, and transform the data. Next, based on user needs and interests, the content push module utilizes the optimization results of ISSA to accurately push educational content, improving the pertinence and effectiveness of education.

## 3 Results

### 3.1 Comparative analysis of ISSA performance

**Figure 7:**
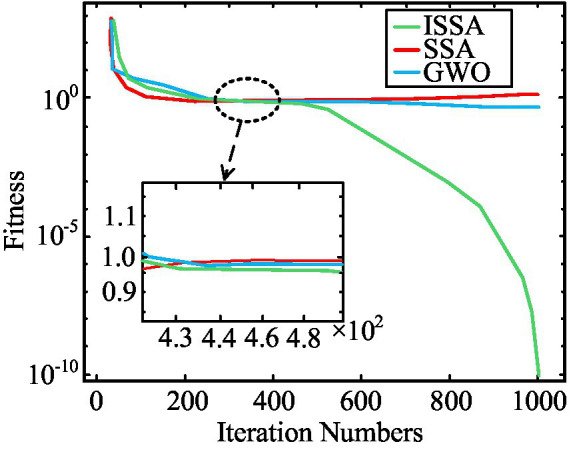
The curve of the optimal fitness value changing with iteration.

To verify the superiority of the ISSA proposed in the study, a series of comparative experiments were conducted. In the above experimental environment, the study used a simulated dataset of public health education resource allocation to verify the performance of the ISSA. This dataset contains 1,000 resource allocation samples, each containing 20 features, used to simulate real public health education resource allocation scenarios. In the data preprocessing stage, missing value filling, outlier handling, and standardization were performed on the data to ensure its accuracy and consistency. To evaluate the performance of ISSA, SSA and GWO algorithms were selected as comparison algorithms for fitness curve comparison. The fitness curves of the three different algorithms working independently with iterative changes are shown in Figure 7.

**Figure 8:**
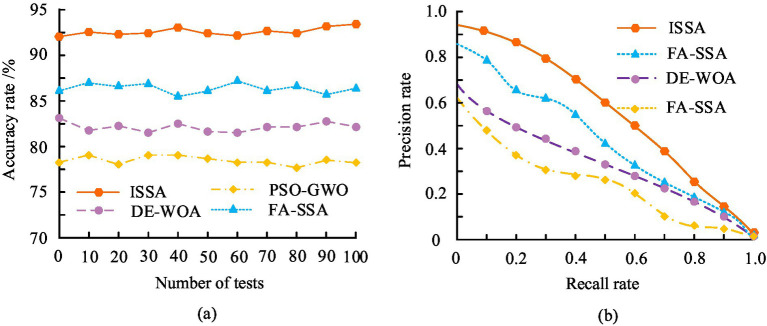
Comparison results of accuracy and PR curves of different algorithms. **(a)** The accuracy results of different models; **(b)** The precision results of different models.

From Figure 7, before reaching 500 iterations, there was not much difference in the fitness values of the three algorithms, and the trend of curve changes remained basically consistent. But after reaching 500 iterations, the fitness value of ISSA showed a significant decrease, while the fitness values of SSA and GWO algorithm showed no significant change. Among them, the average optimal fitness value obtained by ISSA was 1.13 × 10^−6^, with a mini value of 0 and a max value of 0.0586, which was significantly better than SSA and GWO algorithm. In addition, to further demonstrate the superiority of the ISSA over existing optimization algorithms, comparative experiments were conducted with the currently novel DE-WOA, PSO-GWO, and FA-SSA algorithms, with accuracy and PR curve as the comparison indicators. The comparison results are denoted in Figure 8.

**Figure 9:**
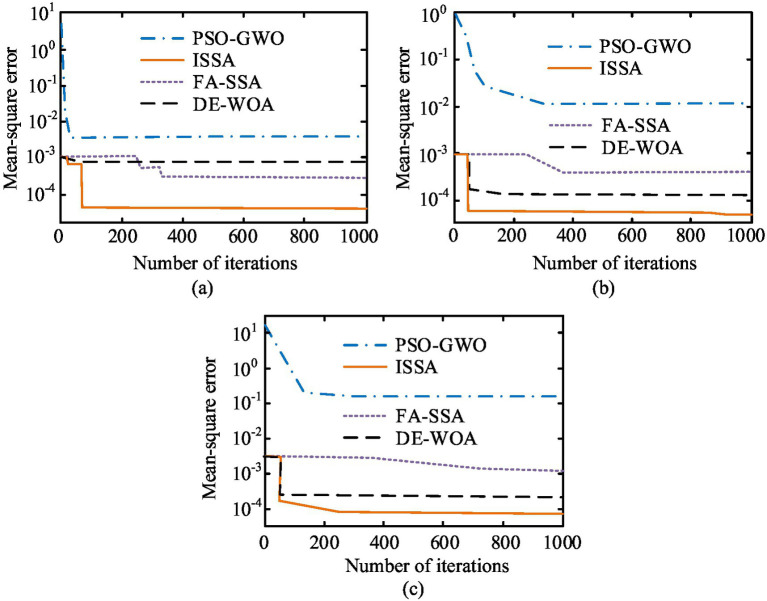
Comparison results of convergence of different algorithms in three data sets. **(a)** The mean square error of different models in the RAD dataset; **(b)** The mean square error of different models in the OBD dataset; **(c)** The mean square error of different models in the MLR dataset.

In Figure 8a, the accuracy curves of the ISSA were higher than those of the comparison algorithms, and the average accuracy of this algorithm was 92.4%, significantly better than the FA-SSA algorithm’s 86.2%, DE-WOA algorithm’s 83.8%, and PSO-GWO algorithm’s 78.3%. In addition, through the accuracy curve, the accuracy fluctuation amplitude of ISSA was lower compared to other algorithms, indicating its higher stability. According to Figure 8b, the PR curve offline areas of ISSA, FA-SSA, DE-WOA, and PSO-GWO were 0.84, 0.72, 0.68, and 0.62, respectively. The larger the area under the PR curve, the better the performance of the algorithm. Therefore, this result indicated that the ISSA proposed in the study had better performance than the compared algorithms. Overall, in terms of accuracy and PR curve dimensions, the ISSA proposed in the study performed better. Afterwards, these four different optimization algorithms were placed on three different datasets to test and compare their convergence. The comparison outcomes are indicated in Figure 9. The three datasets involved in Figure 9 were Resource Allocation Dataset (RAD), Optimization Benchmark Dataset (OBD), and Machine Learning Repository (MLR).

From Figure 9a, in the RAD dataset, the mean square error of all four algorithms decreased with increasing iteration times, and the ISSA converged to the optimal solution first. At this point, its iteration times and mean square error were 41 and 0.00004, respectively, which were better than the comparison algorithms. From Figure 9b, in the OBD dataset, the ISSA proposed by the study was still the first to converge to a stable value, and its optimal mean square error was much smaller than the comparison algorithm. In Figure 8c, the convergence speed of ISSA was not as fast as DE-WOA, but the optimal value after convergence ess better than DE-WOA. The above results indicate that, from the perspective of convergence, the proposed ISSA was significantly superior to the compared FA-SSA, DE-WOA, and PSO-GWO. In summary, the ISSA proposed in the study outperforms the comparative algorithms in all dimensions, so using it to optimize public health education resources will have better results.

### 3.2 Comparative analysis of optimization models for public health education resources

**Figure 10:**
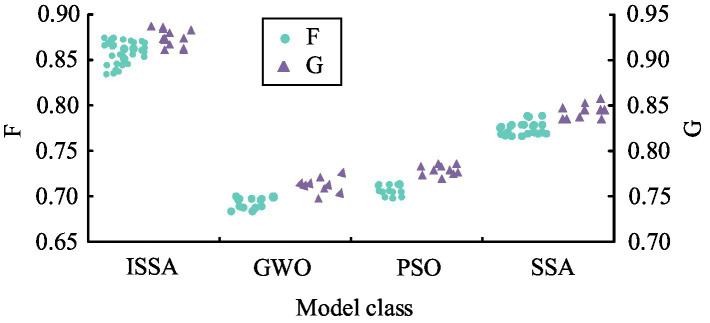
Comparison results of F-value and G-value of the four models.

To verify the actual effectiveness of the public health education resource optimization model proposed in the study, actual cases of public health education resource allocation were selected as experimental subjects to ensure the practicality and reliability of the experimental results. During the experiment, SSA, PSO, GWO, and the proposed ISSA optimization models were applied to optimize and analyze the allocation of public health education resources. By comparing the F-value, G-value, running time, and accuracy of different models, the comprehensive performance of the models in optimizing resource allocation, improving educational effectiveness, and operational efficiency can be reflected. The comparison results of F-value and G-value for each model are shown in Figure 10.

**Figure 11:**
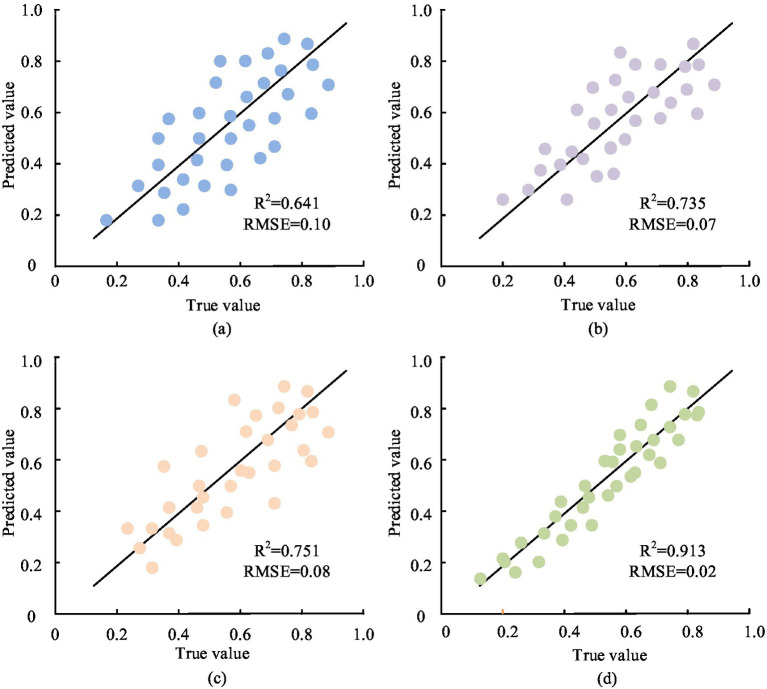
Comparison results of R2 and RMSE values of different optimization models. **(a)** The comparison results of R2 and RMSE values of the ISSA model; **(b)** The comparison results of R2 and RMSE values of the GWO model; **(c)** The comparison results of R2 and RMSE values of the PSO model; **(d)** The comparison results of R2 and RMSE values of the SSA model.

According to Figure 10, the F-values of ISSA, GWO, PSO, and SSA optimization models were concentrated around 0.86, 0.68, 0.72, and 0.78, respectively. In addition, it was found that the G-values of ISSA, GWO, PSO, and SSA optimization models were approximately 0.92, 0.76, 0.77, and 0.84, respectively. The higher the F and G values, the better the optimization effect of the optimization model. Therefore, based on the above results, the overall optimization effect of the ISSA optimization model was better than the three comparison models. In addition, to comprehensively evaluate the efficacy of the optimization model for public health education resources, the R^2^ and root mean square error (RMSE) values of different optimization models were tested and compared. The comparison results are shown in Figure 11.

R^2^ in Figure 11 is an indicator that measures the strength of the linear relationship between the predicted values and the actual values. The higher its value, the better the fitting effect of the model. Similarly, RMSE is used to measure the deviation between predicted values and true values, with smaller values indicating higher prediction accuracy. In Figure 11, the ISSA optimization model proposed in the study had the highest R2 of 0.913, which was 0.178 higher than the R^2^ of the SSA optimization model. This result indicated that the ISSA optimization model had a better fitting effect. In addition, from Figure 11, the RMSE of the ISSA optimization model was also the smallest, at 0.02, significantly lower than the RMSE of the other three comparison models. The above outcomes indicated that the efficacy of the ISSA optimization model was superior to the comparison model. Finally, to further confirm the practical application effect of the ISSA optimization model, this model was applied to the optimization of public health education with three comparative models. The actual optimization effects of each model are denoted in Table 1.

**Table 1:** Comparison of practical application effects of public health education resource optimization model.

Optimization model	The balance of resource allocation after optimization	Percentage increase in educational effectiveness (%)	Run time (seconds)	Increase in user satisfaction (%)
ISSA model	0.89	25.5	118.2	31.4
GWO model	0.75	18.2	150.5	13.1
PSO model	0.70	15.1	183.4	14.8
SSA model	0.68	12.3	204.8	15.6

From Table 1, the ISSA model performed the best in optimizing resource allocation balance, educational effectiveness improvement percentage, running time, and user satisfaction improvement ratio. Among them, the resource allocation balance of the ISSA model reached 0.89, the educational effect improved by 25.5%, the running time was the shortest (118.2 s), and the user satisfaction improvement rate was the highest (31.4%). In contrast, the performance of the other three models on these indicators was not as good as that of the ISSA model, which further validated the superiority of the ISSA model in optimizing public health education resources. In summary, by comparing the actual application effects of the ISSA model with three other optimization models in the allocation of public health education resources, it was found that the ISSA model performed the best in multiple key indicators, verifying its effectiveness and practicality. This result provides strong support for the optimized allocation of public health education resources.

### 3.3 Analysis of the actual application effect of the public health education information platform

**Figure 12:**
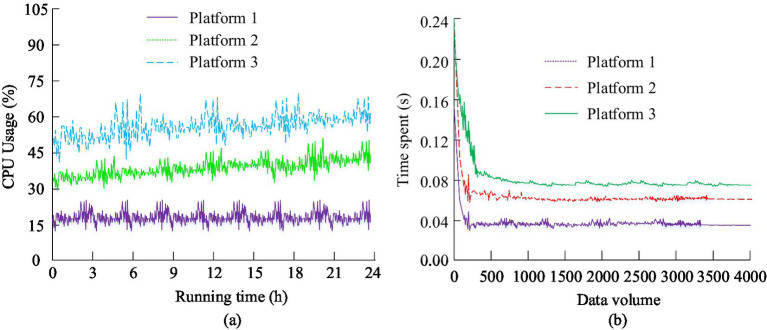
Compares the CPU usage and time consuming of the three platforms. **(a)** CPU usage results of different models; **(b)** Time consumption results of different models.

After completing the comparative analysis of the efficacy of ISSA and the optimization model of public health education resources, the study also analyzed the actual application effect of the proposed public health education information platform. As an important carrier for the allocation and optimization of educational resources, the stability and efficiency of the public health education information platform were crucial. Therefore, the study compared the performance of the proposed education information platform (Platform 1), the public health information platform based on SSA (Platform 2), and the health information platform based on PSO algorithm (Platform 3) in terms of CPU usage and time consumption. The comparison results are shown in Figure 12.

In Figure 12a, the data security model proposed in the study had significantly lower CPU usage than the comparison model, and its usage remained stable at around 18.6%. This indicated that the model could more effectively utilize computing resources and reduce system burden during operation. In Figure 12b, when the data volume was 4,000, the proposed data security model had the lowest time consumption, only 0.037 s, which was much lower than the 0.068 s of the blockchain based data security model and the 0.082 s of the traditional data security model. This further proved the superiority of the model in handling large amounts of data. Finally, to analyze the actual application effect of the proposed public health education information platform, taking public health education in Henan Province as an example, it was applied to optimize public health education. The specific effects were analyzed by comparing various indicators before and after optimization. The data of each indicator before and after optimization for 3 months, 6 months, and 1 year are indicated in Table 2.

**Table 2:** Comparison of indicators before and after the optimization of public health education in Henan Province.

Index	Before optimization	Three months after optimization	Six months of optimization	One year after optimization
Educational resource distribution balance	0.65	0.78	0.82	0.89
Educational content update frequency (times/month)	2	4	6	8
Education coverage (%)	55.3	70.5	81.2	89.6
Student Satisfaction rating (out of 10)	6.8	7.6	8.2	8.9
Public Health Incident Response rating (out of 10)	6.2	7.0	7.8	8.5
Teacher resource utilization rate (%)	61.2	74.2	79.1	84.3
Students’ knowledge of public health (average test score)	62	70	76	82
Number of inter-departmental cooperation projects (units)	5	8	12	18

According to Table 2, after optimizing the proposed public health education information platform, the educational resource allocation balance in Henan Province increased from 0.65 to 0.89, the education coverage rate increased from 55.3 to 89.6%, and the frequency of educational content updates doubled to 8 times per month. The satisfaction of students and the ability to respond to public health incidents have both increased by more than 2 points, and the utilization rate of teacher resources has increased to 84.3%. The students’ mastery of public health knowledge has significantly improved, with an average test score of 84.3 points. The number of cross departmental collaborative projects has significantly increased from 5 to 18. The above results fully demonstrate the significant role of the proposed information platform in improving the effectiveness of public health education, enhancing response capabilities, and promoting cooperation, and further demonstrate the practicality of the public health education optimization strategy based on ISSA and information system technology.

## 4 Discussion and conclusion

The study verified the effectiveness of the optimization strategy for public health education based on ISSA and information system technology through a series of experiments. Firstly, in the performance comparison analysis of the ISSA, the fitness value significantly decreased after 500 iterations, and the average fitness value was better than other compared algorithms. This indicated that the ISSA could converge faster to the global optimal solution when solving the optimization problem of public health education resources. At the same time, the ISSA performed well in both accuracy and PR curve, with an average accuracy of 92.4% and an area under the PR curve of 0.84, further verifying its superiority. This may be because the ISSA, by introducing the theory of best point sets, can more accurately explore the search space, avoid falling into local optima, and thus demonstrate faster convergence speed and higher global search ability in optimizing public health education resources. The experimental results are similar to those of Zhang et al. (33). Secondly, in the comparative analysis of the optimization models for public health education resources, it was found that the ISSA optimization model outperformed other comparative models in indicators such as F-value, G-value, R^2^, and RMSE, especially in terms of resource allocation balance and percentage improvement in educational effectiveness. The resource allocation balance of the ISSA model reached 0.89, the educational effect improved by 25.5%, the running time was the shortest (118.2 s), and the user satisfaction improvement rate was the highest (31.4%). This may be because the ISSA optimization model effectively solved the issue of uneven allocation of public health education resources through iterative optimization of resource allocation strategies, improved resource utilization efficiency, and significantly enhanced educational outcomes and user satisfaction. The above results are consistent with the conclusions obtained by the Gao team’s research on the ISSA optimization model in 2022 (17). In addition, the experimental results of the actual application effect analysis of the public health education information platform showed that the information platform proposed in the study had significantly lower CPU usage than the comparative model and the lowest time consumption. In the practical application of public health education in Henan Province, the platform has significantly improved indicators such as the balance of educational resource allocation, educational coverage, and frequency of educational content updates. At the same time, it has also increased student satisfaction, public health event response capabilities, and teacher resource utilization. These results fully demonstrated the significant role of information platforms in improving the effectiveness of public health education, enhancing response capabilities, and promoting cooperation. This result is consistent with the research outcomes of Kang et al. (34).

The study proposed optimization strategies for public health education by introducing ISSA and information system technology, and verified their effectiveness through experiments. The outcomes denoted that the ISSA significantly outperformed traditional algorithms in terms of performance, providing a scientific basis for optimizing the allocation of public health education resources. The optimization model for public health education resources has achieved significant results in practical applications, significantly improving the balance of educational resource allocation, educational effectiveness, and user satisfaction. Meanwhile, the constructed public health education information platform has also achieved good results in practical applications, providing strong support for the optimization of public health education. Although this study has achieved certain results, there are still shortcomings such as low data quality and narrow application areas. Future research can further explore how to improve data quality, address difficulties in information sharing, and better support the optimization of public health education. Meanwhile, ISSA and information system technology can also be applied to other fields to explore their application effects in different scenarios. Through continuous optimization and improvement, it is expected that this strategy will provide more powerful support for the optimization and development of public health education.

## Data Availability

The original contributions presented in the study are included in the article/supplementary material, further inquiries can be directed to the corresponding author.
